# Application of Transcriptomics for Predicting Protein Interaction Networks, Drug Targets and Drug Candidates

**DOI:** 10.3389/fmedt.2022.693148

**Published:** 2022-03-09

**Authors:** Dulshani Kankanige, Liwan Liyanage, Michael D. O'Connor

**Affiliations:** ^1^School of Computer, Data and Mathematical Sciences, Western Sydney University, Campbelltown, NSW, Australia; ^2^Translational Health Research Institute, Western Sydney University, Campbelltown, NSW, Australia; ^3^School of Medicine, Western Sydney University, Campbelltown, NSW, Australia

**Keywords:** gene expression analyses, bioinformatics, pipeline, gene ontology, protein interaction pathways, drug targets, drugs

## Abstract

Protein interaction pathways and networks are critically-required for a vast range of biological processes. Improved discovery of candidate druggable proteins within specific cell, tissue and disease contexts will aid development of new treatments. Predicting protein interaction networks from gene expression data can provide valuable insights into normal and disease biology. For example, the resulting protein networks can be used to identify potentially druggable targets and drug candidates for testing in cell and animal disease models. The advent of whole-transcriptome expression profiling techniques—that catalogue protein-coding genes expressed within cells and tissues—has enabled development of individual algorithms for particular tasks. For example,: (i) gene ontology algorithms that predict gene/protein subsets involved in related cell processes; (ii) algorithms that predict intracellular protein interaction pathways; and (iii) algorithms that correlate druggable protein targets with known drugs and/or drug candidates. This review examines approaches, advantages and disadvantages of existing gene expression, gene ontology, and protein network prediction algorithms. Using this framework, we examine current efforts to combine these algorithms into pipelines to enable identification of druggable targets, and associated known drugs, using gene expression datasets. In doing so, new opportunities are identified for development of powerful algorithm pipelines, suitable for wide use by non-bioinformaticians, that can predict protein interaction networks, druggable proteins, and related drugs from user gene expression datasets.

## Introduction

One aim of transcriptomic analyses is to accurately predict candidate druggable targets for subsequent—and more laborious—testing of unapproved, approved or repurposed drug candidates within cell and/or animal disease models (1). Accordingly, over the past two decades a range of algorithms have been independently developed to classify and collate gene and proteins from transcriptomic and proteomic data. As outlined below, significant progress has been made in creating and refining algorithms for analysis of transcriptomic data—including microarray and RNA-sequencing data. By understanding the approaches that underpin these algorithms, as well as their individual advantages and disadvantages, new opportunities are identified for development of algorithm pipelines that make prediction of protein networks, and associated druggable proteins/ drugs, more widely accessible.

Broad algorithm categories include algorithms for: gene/protein classification *via* gene ontology (GO) terms; prediction of protein-protein interaction (PPI) networks; and establishment of existing drug/drug target databases.

These tools are often designed to analyse gene or protein lists obtained from high-resolution and/or high-throughput transcriptomic or proteomic data. For example, GO tools are routinely used to analyse transcriptomic data to infer biological functions performed by a cell population. Similarly, PPI tools aim to infer specific cellular functions through prediction and visualisation of protein interaction networks. GO, PPI, and other genomic association algorithms have also been developed to identify druggable targets and associated drugs that might be useful for altering the biology of a cell population (as opposed to algorithms designed for drug target prediction).

In a few instances, multiple algorithms have been organised into an analysis pipeline to provide multiple, correlated outputs from input a single input such as transcriptomics data. Nevertheless, there remains significant scope to develop simplified and broadly accessible algorithm pipelines to predict protein networks, druggable targets, and drugs for subsequent cell- and animal-based studies.

This review therefore begins by examining the key approaches developed for pre-processing and analysis of gene expression data—as relates to protein network and target identification. This includes a review of gene enrichment analysis algorithms, then GO, PPI, and drug/target algorithms. This creates a conceptual framework for understanding current—and future potential—algorithm pipelines for predicting protein networks and druggable targets from gene expression data.

## Algorithms for Gene Enrichment Analysis

Initial steps in analysing gene expression data routinely involve identification of genes/proteins that share a common property, such as being differentially expressed (2). It is important to note that co-expression, and/or co-differential expression, within transcriptomic datasets does not necessarily imply the co-expressed genes are functionally connected. Moreover, the presence of a gene transcript does not always correlate with levels and/or activity of the corresponding protein product (due to multiple layers of post-transcriptional regulation) (3). Nonetheless, co-expression and differential expression have for decades provided useful conceptual paradigms for initial steps in gene expression analyses.

Initial transcriptomic analysis steps—termed Gene Enrichment Analysis—make use of algorithms that access data from gene set libraries—in which individual genes are correlated with specific functional identifiers such as GO terms (4). Inputs required for Gene Enrichment Analysis include one or more lists of interesting genes, together with the species type. Outputs from Gene Enrichment Analysis include information such as GO terms and associated gene groupings, as well as related *p*-values derived by comparing the frequency of the GO term genes in the input list with their frequency in the genome.

Different approaches can be used to perform Gene Enrichment Analysis (Table 1), and these approaches can be categorised into one or more of the following algorithm classes.

**Table 1:** Comparison of gene enrichment analysis tools (white background) and PPI pathway tools (grey background).

**Algorithm/input/output**	**Approach**	**Main advantages**	**Limitations/biases/risks**
SEA: GoStat, BinGO, GOToolBox, GFINDERer, DAVID Input: pre-selected gene list Output: enriched GO terms in a tabular form	Enrichment *p*-value calculated on each term from pre-selected list of interesting gene Enriched terms listed in a tabular format ordered by the enrichment *p*-value	1. Can analyse any gene list generated by high-throughput genomic studies or bioinformatics software packages 2. Simple strategy and output format	1. Relations among terms not always captured as GO term hierarchy not fully captured 2. Quality of pre-selected list of interesting genes could impact the enrichment analysis
GSEA: GSEA, g:Profiler, GOrilla, ADGO Input: list of expressed genes Output: GO terms	Uses the entire gene list from a microarray experiment without any pre-selection	1. No need to pre-select interesting genes 2. Experimental values integrated into *p*-value calculation	1. Difficult to summarise many biological aspects of a gene into a meaningful value when the biological study and genomic platform are complex
MEA: ADGO, GeneCodis3, ProfCom, Ontologizer, DAVID, GoToolBox Input: expressed or differently-expressed genes Output: GO annotations	Inherits basic enrichment calculation from SEA and incorporates extra network discovery algorithms by considering the term-to-term relationships	1.Emphasis on network relationships during analysis	1. Terms or genes without strong inter-relationships could be left out from the analysis
**Algorithm/Input/Output**	**Approach**	**Main advantages**	**Limitations/Biases/Risks**
STRING [Szklarczyk et al. (5)] Input: list of genes or proteins Output: protein interaction network diagram and related text file	Collect, score, and integrate publicly available sources of PPI information, and to complement these with computational network predictions	1. High coverage 2. Ease of use 3. Consistant scoring 4. Many organisms 5. Accessible *via* API 6. Modifiable values, e.g. confidence interval 7. Data from Biocarta, BioCyc, GO, KEGG, and Reactome	1. No information on drugs and druggable targets
KEGG [Kanehisa et al. (6)] Input: list of genes Output: the KEGG network interactions in a text format	Assigns functional meanings to genes and genomes at molecular and higher levels	1. Can upload input gene list	1. Input gene list must be annotated by KEGG Orthology identifiers or K numbers
BioCyc [Karp et al. (7)] Input: database name e.g., homo sapiens Output: network diagrams e.g., genome overview	Web portal combining thousands of genomes	1. Accessible *via* API 2. Sequenced genomes, computationally inferred data, and literature reports 3. Varied query, analysis and visualisation tools	1. Unable to create a network pathway by uploading a gene list
Reactome [Fabregat et al. (8)] Input: species name Output: overview of all Reactome pathways	Tool for discovering functional relationships in gene expression and other data	1. Offer programmatic access to the data	1. Cannot generate PPI networks
Pathway Commons [Rodchenkov et al. (9)] Input: interested gene Output: signalling pathways	Integrated resource of public biological pathways	1. Provides search tools 2. Download pathways 3. Software libraries for pathway investigations 4. Web service for programmatic queries	1.Unable to upload a list of interested genes 2. Cannot generate PPI networks

### Gene Enrichment *via* Singular Enrichment Analysis (SEA)

For SEA, users typically first define a subset of interesting genes; this might include statistically-determining all the expressed genes or a list of genes differentially expressed across two treatment types. The SEA algorithm then analyses the user-defined gene list by (i) determining the frequency of genes in the list associated with a particular grouping (e.g., GO term), and (ii) statistically comparing that frequency with the expected frequency if the genes from that grouping were present by chance. The resulting groups of “enriched” genes, and their associated *p*-values, are then listed sequentially from smallest to largest *p*-value.

If the genome of the species being studied is sufficiently annotated, SEA algorithms can efficiently report gene groupings associated with key biological functions from large input gene lists. Widely-used SEA-based tools include GoStat (10), BinGO (11), GOToolBox (12), GFINDer (13), DAVID (14) etc. While these tools have proven useful, SEA algorithms have two inherent limitations. Firstly, the biological relevance of the output is dependent upon the quality of the user-defined input gene list. Secondly, SEA algorithms do not capture hierarchical relationship between the enriched GO terms identified through the SEA analysis—this can lead to hundreds of GO terms being reported for a single input gene list, which makes it difficult to interpret the biological relevance of the output.

### Gene Enrichment *via* Gene Set Enrichment Analysis (GSEA)

As an alternative approach, GSEA algorithms do not require users to first partition gene expression data into lists of expressed/not-expressed or differentially-expressed genes (as occurs with SEA approaches). Instead, GSEA algorithms use the experimental data obtained for all genes. For example, by comparing the expression signals for all assayed genes on a microarray, GSEA algorithms can determine and then use a single expression fold-change parameter for each gene. This approach can avoid some issues or artefacts related to selection or exclusion of input genes (15). However, for more complex experimental data, the GSEA approach can be disadvantageous. For example, genes that have higher expression fold changes make larger contributions to the GSEA output, but this does not always reflect biological realities (e.g., small changes in transcription can have large effects on cell behaviour). Additionally, complexities in expression for individual genes can now be reliably assessed—such as differential promoter and/or exon usage, or multiple small nuclear polymorphisms. These complexities result in genes having multiple expression profiles, genomic locations, *p*-values, etc. Summarising these complexities into a single parameter value for a gene required by GSEA algorithms can be problematic or not possible. Examples commonly used GSEA approaches include GSEA (15), g:Profiler's g:GOSt tool (16), GOrilla (17), ADGO (18) etc.

### Gene Enrichment *via* Modular Enrichment Analysis (MEA)

A third approach to gene enrichment involves MEA. These algorithms build upon the SEA approach by adding the ability to consider relationships between gene-associated terms such as GO terms (or in some cases, pathway information, protein domains, etc.). Examples include ADGO (18), GeneCodis3 (19), ProfCom (20), Ontologizer (21), DAVID (14) and GoToolBox (12). While the MEA approach can provide improved understanding of expression changes for well-characterised genes, it can inherently bias against genes that are more poorly characterised or that have fewer known relationships. Similar to SEA, the quality of the pre-selected gene list impacts the results of MEA algorithms.

## Algorithms for Construction of Protein Interaction Pathways

While gene enrichment analyses provide useful initial steps for identification of co-expressed genes -and associated grouping of functionally-related genes (e.g., *via* GO terms)—the outputs from gene enrichment algorithms do not include detailed information on protein interactions that might occur within the cell or tissue from which the gene expression data was generated. As a consequence, a range of algorithms have been developed to predict PPI networks using lists of expressed, co-expressed, or differentially-expressed genes. In predicting these PPI networks, it is important to note that gene expression and protein activity are not always correlated, and that protein interactions are not limited to direct physical binding. For example, proteins may also interact: indirectly, by sharing a substance in a metabolic pathway; by regulating each other transcriptionally; or by participating in larger multi-protein assemblies (5).

To provide information on potential PPI in a gene enrichment output, a range of tools have been developed to map interactions based on different levels of evidence—from experimental data to text associations in published literature. Examples of this evidence include experimental evidence (e.g., immunoprecipitation), genomic locations, and cooccurrence reports. The PPI algorithms typically ascribe different levels of confidence for each of these different evidence types to provide ranked output for predicted PPIs. In some instances, prediction of protein interaction networks can be progressed iteratively. For example, by first predicting networks from an input gene list, additional database searching can be performed to suggest additional proteins (not in the original input gene list) for incorporation into the predicted networks—if there is evidence for indicating the new proteins may interact with members of the first predicted network. Key PPI algorithms that have gained wide recognition within the field are briefly reviewed below.

### KEGG Pathways

The Kyoto Encyclopaedia of Genes and Genomes (KEGG), is a widely used tool that integrates both manually curated and computationally generated databases into a single resource. KEGG provides information on genomes, PPI pathways, disease molecular pathophysiology, and drugs with known mechanisms of action. KEGG enables a gene list to be uploaded through an application programming interface (API), however, this function is somewhat limited as it requires the gene list to be annotated with KEGG Orthology identifiers (6).

### BioCyC

The BioCyc database contains genome and predicted metabolic networks for >3,000 organisms. BioCyc enables programmable online access to information including reactions, metabolic pathways, and enzymes (22). BioCyC provides tools to query, analyse, and visualise genome and metabolism data, but does not generate PPI networks from a list of input genes (22).

### Reactome

The Reactome pathway knowledgebase contains non-redundant curated information from other pathway databases such as CheEBI and UniProt (23). It supports API access to investigate unexpected functional relationships in gene expression profiles, however, similar to BioCyc it does not generate PPI networks.

### Pathway Commons

Pathway Commons collates biological pathway information from sources such as BioGRID, HumanCyc, Reactome, NCI/Nature PID, etc. (24). To analyse pathway information, user datasets can be programmatically queried *via* a web service, software libraries and keyword searches. However, it cannot generate protein interaction pathways or upload a list of input genes.

### String

STRING is a pathway database that collects, scores, and integrates public information relating to PPIs from various sources. The basic interaction units in STRING are links between proteins that are known to contribute to specific biologic processes—also known as functional associations. STRING extracts curated data from Biocarta, BioCyc, GO, KEGG, and Reactome (5). STRING has information for >5,000 organisms and >24 million proteins. It provides access through an API and can generate PPI network diagrams that can be user-customised by changing parameters such as the PPI confidence level. While gene lists can be uploaded, there still remain computational challenges including: (i) outputting PPI networks for each individual genes within a gene list (rather than interactions between members of an input gene list); (ii) comparing predicted PPI networks against the set of expressed genes for a tissue; and (iii) iterating these processes for all genes in an input gene list.

## Algorithms for Drug/Target Discovery

A natural progression from having PPI networks predicted from gene expression data is the development of algorithms to correlate proteins in the networks with known small-molecule modulators of protein activity. Toward this end, improvements in genome sequencing have enabled development of pharmacogenetic databases that associate clinical, disease and other annotations (e.g., GO terms) with variations in sequences for particular genes (25). These databases can also link genes as drug targets with related drugs. Key algorithms for mapping proteins to known drugs are briefly reviewed below.

### DrugBank

DrugBank is a highly cited database that combines drug, drug-target, drug action and drug interaction information—but not disease-related or prescribing details. The database is updated daily and is accessible *via* an API, but does not allow users to upload gene list of interest (26).

### PharmGKB

The Pharmacogenomics Knowledgebase (PharmGKB) is a widely used resource containing information curated from other databases including PubMed, DrugBank and the human small nucleotide polymorphism database, dbSNP. The main advantages of PharmGKB are it collates drug targets, drugs, and corresponding disease information in one platform, while providing access *via* an API (25).

### ChEMBL

ChEMBL is an open access online database that extracts data from medicinal chemistry journals and integrates them with information on approved drugs. ChEMBL also exchanges data with other databases such as PubChem and the pharmacokinetics database, BindingDB. The data can be accessed using web services and in downloadable formats, although it does not provide integrated PPI/drug/target outputs (27).

### TTD

The Therapeutic Target Database (TTD) provides information about drug targets, drugs, and related disease conditions. It extracts data from pathway databases including KEGG, MetaCyc, Reactome, and Wikipathways. TTD integrates drug/target/disease outputs, however, users can not upload gene lists or generate PPI networks. TTD also does not provide access *via* an API (28).

### DGIdb

The drug–gene interaction database (DGIdb) integrates drug–gene interactions and potential druggable genes. It consolidates data from other databases such as GO, DrugBank, PharmGKB, the TTD and the National Cancer Institute (NCI). Users can upload gene lists to identify any associated drugs. However, similar to DrugBank, it does not capture related disease data (29).

### DisGeNET

The disease-gene network database, DisGeNet, contains information on genes and their variants related only to human diseases. DisGeNET extracts data from different scientific literature and consolidates them using text mining tools. Data on DisGeNet database can be downloaded in many file formats, and accessed through an API (30).

### PHAROS

PHAROS aims to provide insight on the druggable genome. It contains information on all human protein targets (both well- and poorly-characterised targets) including protein expression data, GO terms, disease associations and drug target interactions. While API access is possible, it is highly time-consuming as only one gene can be submitted at a time (31).

### GPSnet

The genome-wide positioning systems network (GPSnet) is a recent algorithm focused on identifying cancers for which approved or investigational drugs might be repurposed to provide candidate new treatment options. GPSnet functions in two steps: (i) it analyses sequencing information from 15 cancer types (from ~5,000 patients); and then (ii) implements a GSEA algorithm and network proximity methods to identify candidate drugs for the targets identified in the first step (32). While this provides a powerful approach to identifying candidate new drug treatments, the information is not accessible through an API and the database currently focuses only on cancers.

## Algorithm Pipelines for Predicting Druggable Targets/Drugs From Expression Data

As outlined above, a large range of algorithms have been developed for analysis of gene expression data. To date, only a small number of algorithm pipelines have been developed to progress from gene enrichment analysis, through GO analysis and protein network prediction, to drug target identification (Table 2). Of these pipelines, PHAROS, DGIdb and GPSnet are the most well-developed. PHAROS can generate PPI networks, identify drug targets and associated drugs when a list of genes is provided. However, PHAROS is human-specific and the API does not allow multiple genes as an input. Also, the data output is not grouped by GO terms, which would facilitate rapid assessment of PPI networks and associated drugs based on key biological processes or molecular functions. DGIdb integrates data from many other drug databases, and an input gene list can be uploaded. However, it does not capture disease information which is a significant disadvantage. GPSnet, a more recently developed algorithm, can perform GSEA and generate protein interaction networks. Unfortunately, it currently focuses only on cancer specific diseases and does not provide a free online too to access its data *via* an API.

**Table 2:** Comparison of drug/target discovery tools.

**Algorithm/Input/Output**	**Approach**	**Main advantages**	**Limitations/biases/risks**
PharmGKB [Barbarino et al. (25)] Input: list of genes or proteins Output: druggable genes	An organised knowledgebase containing genetic, clinical, and cellular phenotype knowledge networks	1. Data curated from PubMed, DrugBank, dbSNP 2. Accessible *via* API	1. Does not have the facility to input a list of genes and generate pathways
DrugBank [Wishart et al. (26)] Input: single drug target Output: drug information	Bio-/chemo-informatics resource that combines drug and drug target information	1. Updated daily	1. Cannot input a gene list or generate PPI networks
ChEMBL [Gaulton et al. (27)] Input: single drug target Output: drug information	An open large-scale bioactivity database combining molecule, target, and drug data	1. Provide web services for programmatic access to limited to specific queries	1. Does not provide integrated PPI/drug/target outputs
TTD [Chen et al. (4)] Input: list of genes Output: related drug target and disease information	Provides details on known therapeutic nucleic acid and protein targets, associated disease conditions, pathway information, and drug/ligand details	1. Can search *via* target name, drug name, disease name and so on 2. Pathways use KEGG, MetaCyc, Reactome, Wikipathways	1. Cannot create pathways *via* input gene lists
DisGeNET [piñero et al. (33)] Input: list of genes Output: summary/evidence of gene-disease associations	Comprehensive archive of genes and variants associated to human diseases. Collection of data on genotype-phenotype relationships from several of the most popular resources in this area	1. Database can be searched by target or drug/ligand names 2. Genes and variants associated to human diseases	1. No PPI networks for input genes before drug target determination 2. Only focuses on genes/variants involved in human diseases
DGIdb [Cotto et al. (29)] Input: list of genes or proteins Output: associated drugs	A collection of drug–gene interactions and gene druggability information	1. Combines drug–gene interactions and possible druggable genes 2. Combines data from NCI, PharmGKB, TTD, GO, DrugBank etc 3. Can upload gene list	1.Unable to generate the PPI network 2.Does not capture disease data
PHAROS [Nguyen et al. (31)] Input: interested list of genes or proteins Output: gene ontology terms, pathways, drugs, diseased grouped by the input gene	Is a web interface which presents data from the Target Central Resource Database (TCRD) which collates many heterogeneous gene/protein datasets	1. Considers all human protein targets 2. Information on understudied targets 3. Offer programmatic access to the data 4. Consists of protein expression data, disease and phenotype associations, bioactivity data, drug target interactions 5. Includes GO terms	1. Unable to upload gene lists 2. Data output is not grouped based on GO terms (thus does not enable rapid assessment of PPI networks and associated drugs based on key biological processes or molecular functions performed by the cell of interest) 3. Human-specific
GPSnet [Cheng et al. (32)] Input: list of cancer genes Output: drug target network	Network based, integrated algorithm for cancer related diseases and approved or investigational drugs	1. Drug-target networks 2. Uses GSEA algorithm in gene enrichment	1. Focus only on cancer-specific diseases 2. No free API access

*Comparison of drug/target discovery tools (most developed pipelines shown in grey)*.

Given the capabilities and limitations with the above-mentioned algorithms, there is a need and opportunity to develop an algorithm pipeline that: (i) enables users to upload gene expression data as an input (either expressed or differentially-expressed gene lists); (ii) groups genes based on their GO terms; (iii) creates additional outputs for each gene consisting of visualised PPI networks that include identified druggable targets and associated drugs; and (iv) has the potential to identify new pathways, and thus druggable target discovery, through modification of PPI network prediction parameters (for example, by modifying related parameters used by STRINGdb).

## Conclusion

As reviewed here, much progress has been made in developing a wide variety of algorithms for gene expression analyses. However, a gap in the field exists for algorithm pipelines that combine all these important analysis tools into unified and simplified packages suitable for broad use by non-bioinformaticians. These algorithm pipelines should allow users to upload gene expression datasets from human and non-human species. They should also provide integrated outputs including GO terms, protein network predictions, and drug target, drug and disease information. Astute combination and modification of existing algorithms could address this gap. Once realised, these new algorithm pipelines could accelerate identification and prioritisation of drug targets, and associated drugs—in order to minimise the time and labour costs associated with testing unapproved, approved or repurposed drug candidates in cell and animal models of disease.

## Author Contributions

All authors were involved in outlining, writing, and reviewing the manuscript.

## Conflict of Interest

The authors declare that the research was conducted in the absence of any commercial or financial relationships that could be construed as a potential conflict of interest.

## Publisher's Note

All claims expressed in this article are solely those of the authors and do not necessarily represent those of their affiliated organizations, or those of the publisher, the editors and the reviewers. Any product that may be evaluated in this article, or claim that may be made by its manufacturer, is not guaranteed or endorsed by the publisher.
